# Supplementary material for: Structural and Functional Characterization of Orcokinin B-like Neuropeptides in the Cuttlefish (Sepia officinalis)
Source: Mar Drugs. 2022 Aug 4;20(8):505. doi: 10.3390/md20080505 (PMC9410093; doi:10.3390/md20080505)
Supplement: Supplementary file 1 [file marinedrugs-20-00505-s001.zip › marinedrugs-1773385-supplementary.pptx]

## Slide 1
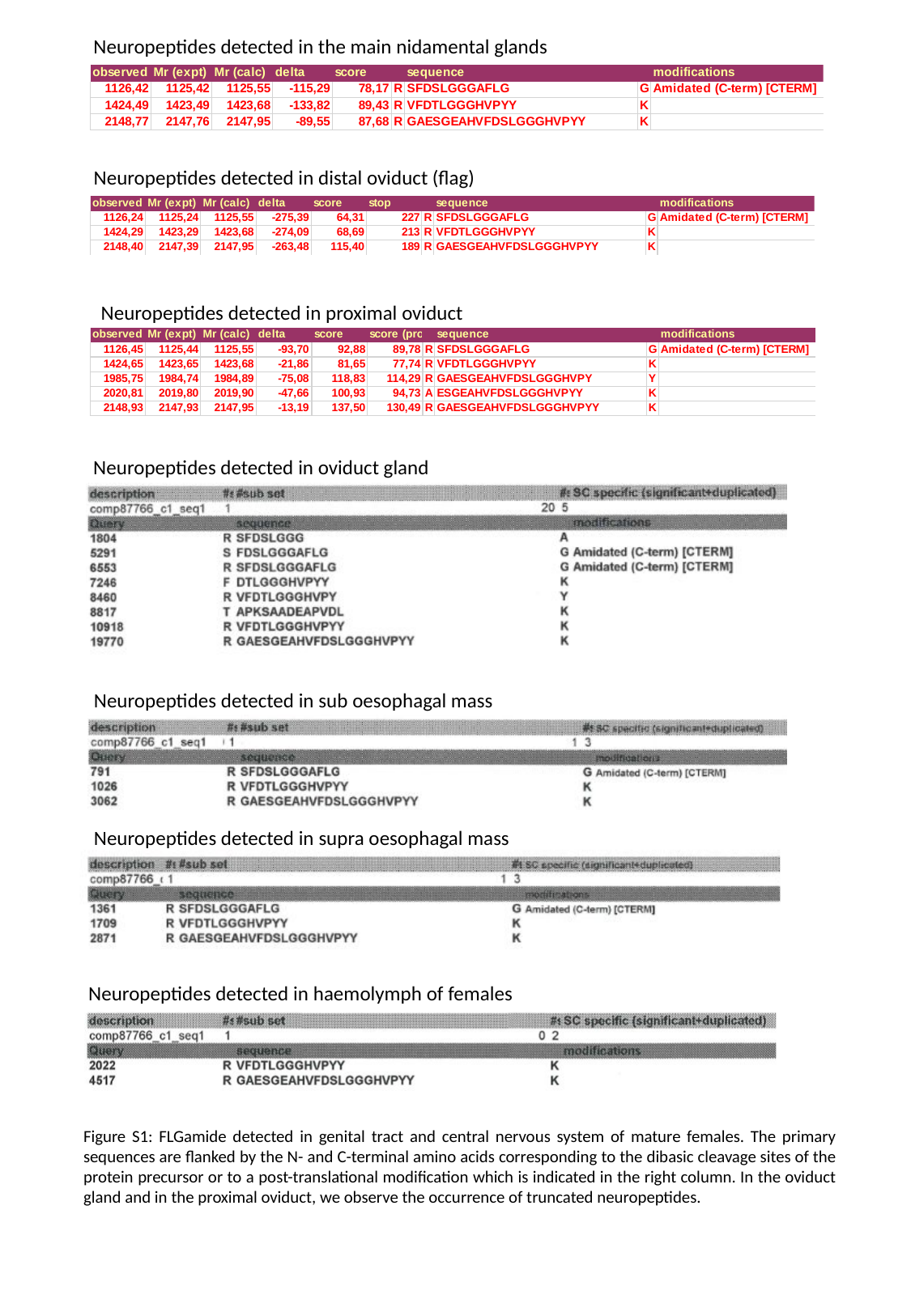

Neuropeptides detected in the main nidamental glands
Neuropeptides detected in distal oviduct (flag)
Neuropeptides detected in proximal oviduct
Neuropeptides detected in oviduct gland
Neuropeptides detected in sub oesophagal mass
Neuropeptides detected in supra oesophagal mass
Neuropeptides detected in haemolymph of females
Figure S1: FLGamide detected in genital tract and central nervous system of mature females. The primary sequences are flanked by the N- and C-terminal amino acids corresponding to the dibasic cleavage sites of the protein precursor or to a post-translational modification which is indicated in the right column. In the oviduct gland and in the proximal oviduct, we observe the occurrence of truncated neuropeptides.
